# Visuospatial working memory in behavioural variant frontotemporal dementia: a comparative analysis with Alzheimer's disease using the box task

**DOI:** 10.1007/s00415-024-12406-0

**Published:** 2024-05-07

**Authors:** David Foxe, Muireann Irish, James Carrick, Sau Chi Cheung, Her Teng, James R. Burrell, Roy P. C. Kessels, Olivier Piguet

**Affiliations:** 1 School of Psychology, The University of Sydney, Sydney, Australia; 2 Brain and Mind Centre, The University of Sydney, 94 Mallett St, Sydney, NSW 2006 Australia; 3 Neuropsychology Unit, Royal Prince Alfred Hospital, Sydney, Australia; 4 Concord Medical School, The University of Sydney, Sydney, Australia; 5 Donders Institute for Brain, Cognition and Behaviour, Radboud University, Nijmegen, The Netherlands; 6 Vincent Van Gogh Institute for Psychiatry, Venray, The Netherlands; 7 Radboud University Medical Center, Radboudumc Alzheimer Center, Nijmegen, The Netherlands

**Keywords:** Frontotemporal dementia, Alzheimer’s disease, Cognitive assessment, Visuospatial working memory

## Abstract

**Objective:**

This study investigated the visuospatial working memory profiles of behavioural variant frontotemporal dementia (bvFTD) and Alzheimer’s disease (AD) using a novel computerised test of visuospatial working memory: the Box Task.

**Methods:**

Twenty-eight bvFTD and 28 AD patients, as well as 32 age-matched control participants were recruited. All participants completed the Box Task and conventional neuropsychological tests of working memory, episodic memory, and visuospatial function.

**Results:**

Both the bvFTD and AD groups exhibited significantly more Box Task between-search errors than the control group across all set sizes. Notably, the AD group demonstrated a significantly higher error rate compared to the bvFTD group. Regression analysis revealed that whilst episodic memory impairment significantly predicted Box Task error performance in AD, this was not the case for bvFTD. Additionally, a noticeable trend was observed for attention in predicting Box Task errors in both bvFTD and AD groups. The Box Task demonstrated high utility in differentiating between bvFTD and AD, with a decision tree correctly classifying 82.1% of bvFTD patients and 75% of AD patients.

**Conclusions:**

Our findings reveal significant visuospatial working memory impairments in bvFTD, albeit of lesser severity compared to disease-matched AD patients. The Box Task, a novel measure of visuospatial working memory, proved effective in differentiating between bvFTD and AD, outperforming many traditional neuropsychological measures. Overall, our findings highlight the utility of assessing visuospatial memory when differentiating between bvFTD and AD in the clinical setting.

**Supplementary Information:**

The online version contains supplementary material available at 10.1007/s00415-024-12406-0.

## Introduction

The memory profile of the behavioural variant of frontotemporal dementia (bvFTD) has been a subject of debate [1–3]. The current consensus criteria for bvFTD describe a neuropsychological profile of relatively intact episodic memory and visuospatial abilities in the context of marked executive dysfunction [4]. Recent research, however, demonstrates the presence of significant episodic memory disturbances in the early stages of bvFTD [3, 5–7]; similarly, visuospatial deficits have also been documented [8, 9]. As such, ambiguity remains surrounding the early neuropsychological profile of bvFTD [1, 2] prompting a growing interest in understanding how cognitive processes, such as visuospatial working memory, are impacted [3, 10].

In contrast to bvFTD, Alzheimer's disease (AD) is characterised by prominent deficits in both memory and visuospatial abilities [3, 7, 11, 12]. Extensive literature on AD emphasises the disproportionate impairment of visuospatial memory and underscores its utility in differentiating AD from other dementias, including bvFTD [3, 13]. There has, however, been less emphasis in exploring whether visuospatial *working* memory could serve as a clinical marker for distinguishing AD from bvFTD [10, 14]. Visuospatial working memory involves the capacity to temporarily store and manipulate visual and spatial information in mind (i.e., over a few seconds), and is crucial for forming longer-term memories [15, 16]. Whether alterations in visuospatial working memory could serve as a distinctive clinical marker for discriminating between bvFTD and AD remains unclear [10, 14].

In this study, we examined the visuospatial working memory profiles of individuals with bvFTD and AD using a novel computerised test—the Box Task [17]. The Box Task is an associative working memory task that involves binding information between objects and their spatial location, a process that is particularly impaired in AD [17–19]. Compared to conventional visuospatial memory tests, the Box Task investigates different dimensions concurrently, such as error types, efficiency of strategy search, and provides graded levels of impairment. Based on previous literature [3, 7, 10, 13, 18, 19], we hypothesised that both bvFTD and AD would demonstrate visuospatial working memory deficits, relative to healthy participants. Importantly, however, we predicted that AD patients would perform worse on the Box Task relative to bvFTD patients. To identify potential mechanisms driving Box Task performance, we also assessed visuospatial, memory, and non-verbal abilities. We hypothesised that visuospatial memory impairment would largely predict Box Task error performance in AD, while attentional and executive impairment would predict error performance in bvFTD [10]. To evaluate its diagnostic utility, we compared the Box Task to other cognitive measures typically used to distinguish between bvFTD and AD. Finally, we provide a Box Task decision tree to assist clinicians in using this tool in the differential diagnosis of bvFTD and AD.

## Methods

### Participants

Fifty-six patients diagnosed with dementia (probable AD: 28, probable bvFTD: 28) and 32 healthy controls were recruited between September 2015 and December 2020 from FRONTIER, the younger-onset dementia research clinic based at the Brain and Mind Centre at the University of Sydney, Australia. Patients underwent a comprehensive clinical evaluation with an experienced neurologist, a neuropsychological assessment, and a structural brain MRI scan. The clinical assessment and MRI scan occurred within 3 months of the neuropsychological assessment. Diagnosis was established according to current clinical diagnostic criteria for probable bvFTD [4] and probable ‘amnestic’ (i.e., typical) AD [11]. Disease duration was characterised as the time (years) of diagnosis from the onset of symptoms as described by the carer. Patients with non-amnestic presentations of AD (e.g., ‘executive’ AD, ‘language’ AD [incl. logopenic progressive aphasia], ‘visuospatial’ AD [incl. posterior cortical atrophy]) were excluded [11, 20, 21]. Importantly, Box Task performance was not included in the diagnostic process to avoid circularity. Exclusion criteria for all participants (i.e., patients and control participants) included concurrent psychiatric diagnosis, presence of other dementias or neurological syndromes, traumatic brain injury, history of alcohol or substance abuse, poor English proficiency, and/or absence of a reliable informant. Participants were excluded if they scored < 50/100 (for dementia patients) or < 88/100 (for controls) on the Addenbrooke’s Cognitive Examination-III [ACE-III: 22].

All participants or the person responsible provided written informed consent in accordance with the Declaration of Helsinki. The South Eastern Sydney Local Health District, the University of New South Wales, and the University of Sydney human ethics committees approved the study.

### General cognition and dementia severity assessment

All participants completed the ACE-III as an index of overall cognitive function [22, 23] and underwent a series of cognitive tests measuring the following cognitive constructs: (i) short-term memory (Spatial and Digit Span forward) [24]; (ii) working memory (Spatial and Digit Span backward); (iii) attention (ACE-III attention subdomain; Trail Making Test Part A) [25]; (iv) executive function (Trail Making Test Part B); (v) episodic memory (ACE-III memory subdomain; Rey Complex Figure Test [RCFT] 3-min recall [26]); (vi) language skills (confrontation naming, single-word repetition, and semantic association subtests of the Sydney Language Battery [27]); (vii) verbal fluency (ACE-III fluency subdomain); and (viii) visuospatial/constructional ability (ACE-III visuospatial subdomain; RCF copy trial [26]).

In addition, spouses, relatives, or carers of patients completed the Disability Assessment for Dementia (DAD [28]). The DAD is an informant-based measure that assesses the patient’s level of functioning in activities of daily living. The total DAD score is reported as a percentage of remaining ability, with lower values representing poorer day-to-day functioning.

### The box task

**Fig. 1:**
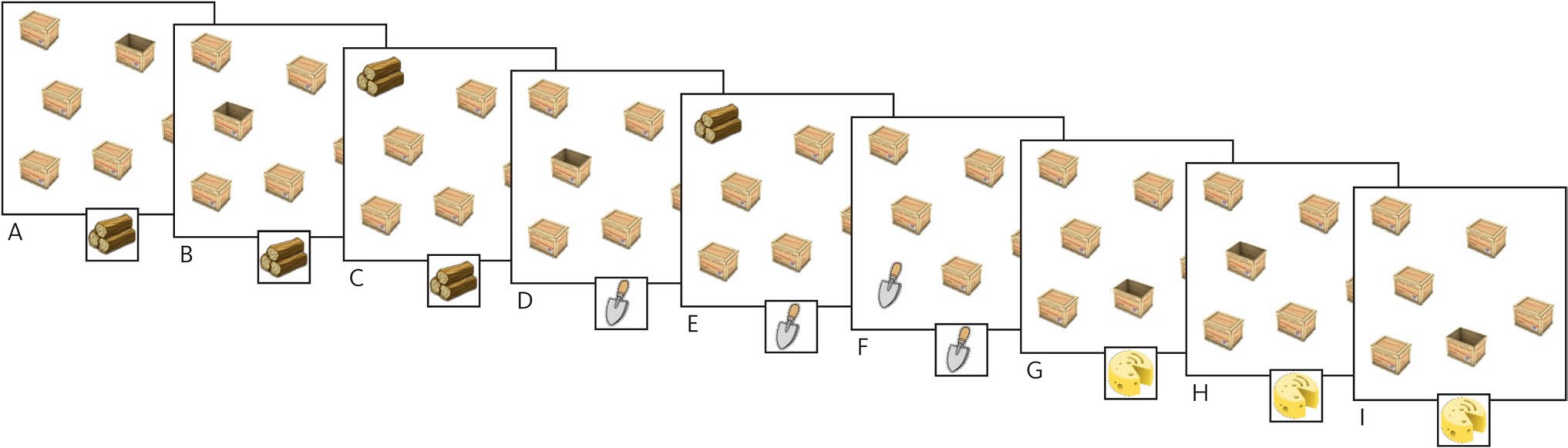
Schematic overview of the Box Task showing a trial run with a set size of 6. The participant has to search for the target object (i.e., wood in **A**–**C**). When the target object is found, the participant has to search for the next target object (i.e., spade and cheese in **D**–**I**). A within-search error is made when the participant returns to a box that was already found to be empty in that search. This error is displayed in **I**. A between-search error is made when the participant opens a box that already contained an object from a previous search. This error is displayed in **E**

The Box Task is a novel computerised visuospatial working memory task in which pictures of closed boxes are presented at various locations on a computer screen, with increasing set sizes (4, 6 and 8 boxes) (Fig. 1; see Kessels and Postma [17] for information on this task). Briefly, participants are instructed to find a hidden target object by ‘opening’ the boxes on the screen one by one. When a target is found, a new target object is presented that has to be searched. Participants are instructed that the previously found object will remain hidden in its box. Thus, participants are not only required to remember which boxes they recently searched but also which boxes contain previous targets. When all target objects are found, a new trial with a new spatial layout and an increased number of boxes starts. Thus, a trial consists of a set of to-be-searched boxes of a given set size and a given spatial layout in which multiple objects have to be found successively; a search refers to finding the hidden location of the current object. The task includes two practice trials that contain three boxes followed by two trials with 4, 6, then 8 boxes (i.e., two trials at the 4-box set size, two trials at the 6-box set size, etc.). There is no time limit imposed but participants are encouraged to respond within a reasonable time (i.e., within a few seconds).

Two error scores were computed from the Box Task (Fig. 1). First, *between-search errors* were calculated, denoting when participants returned to a box containing a target previously encountered from a previous search within that trial. This score reflects the ability to maintain object–location information for periods of time ranging from a few seconds to 1–2 minutes. Second, *within-search errors* were calculated, denoting when participants returned to a box that had already been opened within the current search (i.e., returning to a box that was already found to be empty during that search). This score reflects the ability to keep track of recently searched locations and captures short-term retention of visuospatial information. The time (seconds) to find the hidden location of each object was also recorded. Finally, a normalised Levenshtein edit distance score was computed as a measure of ‘search path strategy’—that is, the participant’s ability to adopt an efficient strategy to complete the task [29]. The normalised Levenshtein edit distance score computes the similarity/difference between two search paths. Briefly, it compares the current search path with the one from the previous search, minus the target from the previous search, resulting in a score ranging from 0–1. In this study, lower distance scores represent a more proficient strategy to complete the task.

### Statistical analyses

Data were analysed using IBM SPSS Statistics, 26.0 (SPSS Inc., Chicago, Ill., USA) and figures were created with GraphPad Prism 9 (GraphPad Software, San Diego, CA, USA). Distribution of demographic, neuropsychological, and behavioural data were inspected using Shapiro–Wilks tests. Normally distributed variables were compared across all groups (i.e., bvFTD, AD, controls) using one-way ANOVAs followed by Sidak post hoc tests; *T* tests were used to compare normally distributed variables across two groups (bvFTD, AD). Variables not normally distributed were analysed using Kruskal–Wallis ANOVA followed by Dunn-Bonferroni post hoc tests. Chi-square tests (χ^2^) were used to analyse categorical measures (e.g., sex).

Three separate Generalised Estimating Equations (GEE) regression models were used to analyse the Box Task between-error, within-error, and strategy variables. The GEE factors included trial (i.e., two per set size) and set size (i.e., 4, 6, 8 boxes) nested under group (i.e., bvFTD, AD, controls). The participant was included as the subject variable. A Poisson distribution was selected for the between-error GEE as the between-error variable reflected count data, whereas a linear distribution was selected for the within-error and strategy GEEs as these variables reflected scale numeric data. Null hypotheses of no change in all fixed effects were tested. Parameter estimates of the overall group and group by box set size were derived. For the time variable, a set size (i.e., 4, 6, 8 boxes) × group repeated measures mixed model ANOVA was conducted, as the 4-box Trial 1 time was not available due to a computing error (thus GEE modelling could not be conducted). The 4-box Trial 2-time score (only) was analysed along with the respective mean (i.e., average of Trial 1 and 2) for 6-box and 8-box set size time scores. For both GEE and repeated measures mixed model analyses, post hoc pairwise comparisons of groups were analysed using least-significant difference (LSD), with the false discovery rate controlled using the Benjamini–Hochberg procedure [30]. The false discovery rate *Q* was set at 0.05.

We evaluated the relative contribution of basic visuospatial ability, memory (i.e., short-term, working, episodic), attention, and executive functioning to the Box Task 6-box between-search error scores in bvFTD and AD by conducting two separate multiple regression analyses (enter method). The Box Task 6-box between-search error measure was selected as it was previously shown to evoke an optimal level of cognitive demand and complexity compared to the 4- and 8-box set sizes [18, 19]. For both analyses, the 6-box between-search error score (average error scores over two trials) was the dependent variable and the cognitive test measures (ACE-III visuospatial, WMS-III Spatial Span [forward, backward], RCF copy and 3-min recall, Trails A and B [time and errors]) were the independent variables. The standardised beta coefficient values for each regression model were inspected to determine the contribution (i.e., magnitude of effect) of each cognitive ability on between-search error scores. For executive function, the Trails B minus A time difference score was used rather than Trails B time score due to collinearity concerns with Trails A time.

We inspected the area under the receiver operating characteristic curve (AUC) for all Box Task variables (i.e., average performance over two trials at 4, 6, and 8 box set size) as compared to other cognitive tests of interest to determine the most sensitive measures to differentiate bvFTD from AD patients. Statistical significance for all AUC analyses was set at *p* < 0.05.

Finally, the chi-square automatic interaction detector (CHAID) method was used to create a decision tree to classify bvFTD versus AD patients based on their ACE-III total (/100) performance (n.b., the first variable forced into the model) and between- and within-search error scores (total score of trial 1 and 2) at each set size (i.e., 4, 6 and 8 boxes). No data from control participants were included in this analysis. The rationale for the variables included in the CHAID model is provided in the Supplementary Material.

## Results

### Demographics

Groups were comparable for sex (*p* = 0.169) and age (*p* = 0.069) but not for years of education (*p* = 0.019), with the control group having more years of education than the bvFTD group (*p* = 0.020) (Table 1). Patient groups did not differ in disease duration (*p* = 0.283) or functional capacity (i.e., DAD total; *p* = 0.936). An overall group difference was present for general cognitive functioning on the ACE-III total (*p* < 0.001), driven by better performance in controls relative to the patient groups (all *p* values < 0.001). Importantly, the bvFTD and AD groups were comparable on the ACE-III total (*p* = 0.060).

**Table 1:** Demographic characteristics and cognitive performance of study participants

	AD	bvFTD	Controls	Group effect (F or H)	*p*	Post hoc test^#^
Sex (m: f)	16:12	19:9	14:18	3.552†	.169	
Age (y)	66 (8.8)	62.4 (8)	66.7 (5.3)	2.764	.069	
Education (y)	13.2 (2.8)	11.8 (2.6)	13.7 (2.4)	4.13	.019	bvFTD < Controls
Disease duration (y)	3.9 (1.9)	5.0 (2.5)	–	1.177	0.283	
DAD total (100)	76.4 (18.2)	51.5 (18.0)	–	0.007	0.936	
ACE-III total (100)	68.3 (10.5)	74 (11.7)	95.1 (3)	74.776	< 0.001	AD, bvFTD < Controls
Visuospatial ability and episodic memory						
ACE-III visuospatial (16)	12.5 (3.4)	14.3 (1.5)	15.4 (0.9)	13.717	< 0.001	AD < bvFTD, Controls
RCF copy (36)	24.9 (8.5)	26.2 (7.2)0	31 (4.1)	11.367^	0.003	AD, bvFTD < Controls
RCF copy time (secs)	282.2 (141.3)	202.4 (123.2)	182.5 (68.7)	11.757^	0.003	AD > bvFTD, Controls
RCF 3-min recall (36)	3 (3.4)	8.3 (6.8)	16.7 (5)	47.878^	< 0.001	AD < bvFTD < Controls
Visuospatial short-term and working memory						
Spatial Span forward (16)	5.5 (2.2)	6.4 (2.1)	8 (1.5)	12.741	< 0.001	AD, bvFTD < Controls
Spatial Span backward (16)	4.3 (2.5)	5.9 (2.4)	7.5 (1.8)	15.36	< 0.001	AD < bvFTD < Controls

Values are mean ± standard deviation

Number of missing values: Education = 1 control; DAD = 1 AD, 3 bvFTD; RCF copy = 3 AD; RCF copy time = 3 AD, 1 bvFTD; RCF 3-min recall = 3 AD

*ACE-III* Addenbrooke’s Cognitive Examination–Third edition, *AD* Alzheimer’s disease, *bvFTD* behavioural variant frontotemporal dementia, *DAD* Disability Assessment for Dementia, *Disease duration* time (years) of diagnosis from the onset of symptoms as described by the carer, *f* female, *m* male, *RCF* Rey Complex Figure, *Spatial Span* Spatial Span from the Wechsler Memory Scale—Third Edition, *y* years

†χ^2^ test

^Kruskal–Wallis ANOVA

#Sidak corrected for parametric tests, Bonferroni corrected for non-parametric tests

### Cognitive profiles of participants

Both dementia groups showed the prototypical cognitive profiles on neuropsychological testing (see Supplementary Material). Notably, the bvFTD group performed comparably to the control group on a basic measure of visuospatial ability (ACE-III visuospatial, *p* = 0.137), but demonstrated difficulties on a more complex visuoconstructional task (RCF copy score, *p* = 0.020). Visuospatial episodic memory (RCF 3-min recall, *p* < 0.001), and visuospatial short-term (Spatial Span forward, *p* = 0.007) and working memory (Spatial Span backward, *p* = 0.018) were also impaired in bvFTD relative to controls. In contrast, the AD group displayed impairments on all measures of visuospatial ability (ACE-III visuospatial, *p* < 0.001; RCF copy score, *p* = 0.003), visuospatial episodic memory (RCF 3-min recall, *p* < 0.001) and visuospatial short-term and working memory (Spatial Span forward, *p* < 0.001; Spatial Span backward, *p* < 0.001) relative to the control group (Table 1).

Direct comparisons between patient groups indicated greater visuospatial dysfunction (ACE-III visuospatial, *p* = 0.008), and visuospatial episodic (RCF 3-min recall, *p* = 0.001) and working memory (Spatial Span backward, *p* = 0.031) impairment in AD compared with bvFTD. Of note, the AD group was significantly slower than the bvFTD and the control groups at completing the RCF copy task (RCF copy time, both *p* values ≤ 0.036).

## Box Task

### Between-search errors

**Fig. 2:**
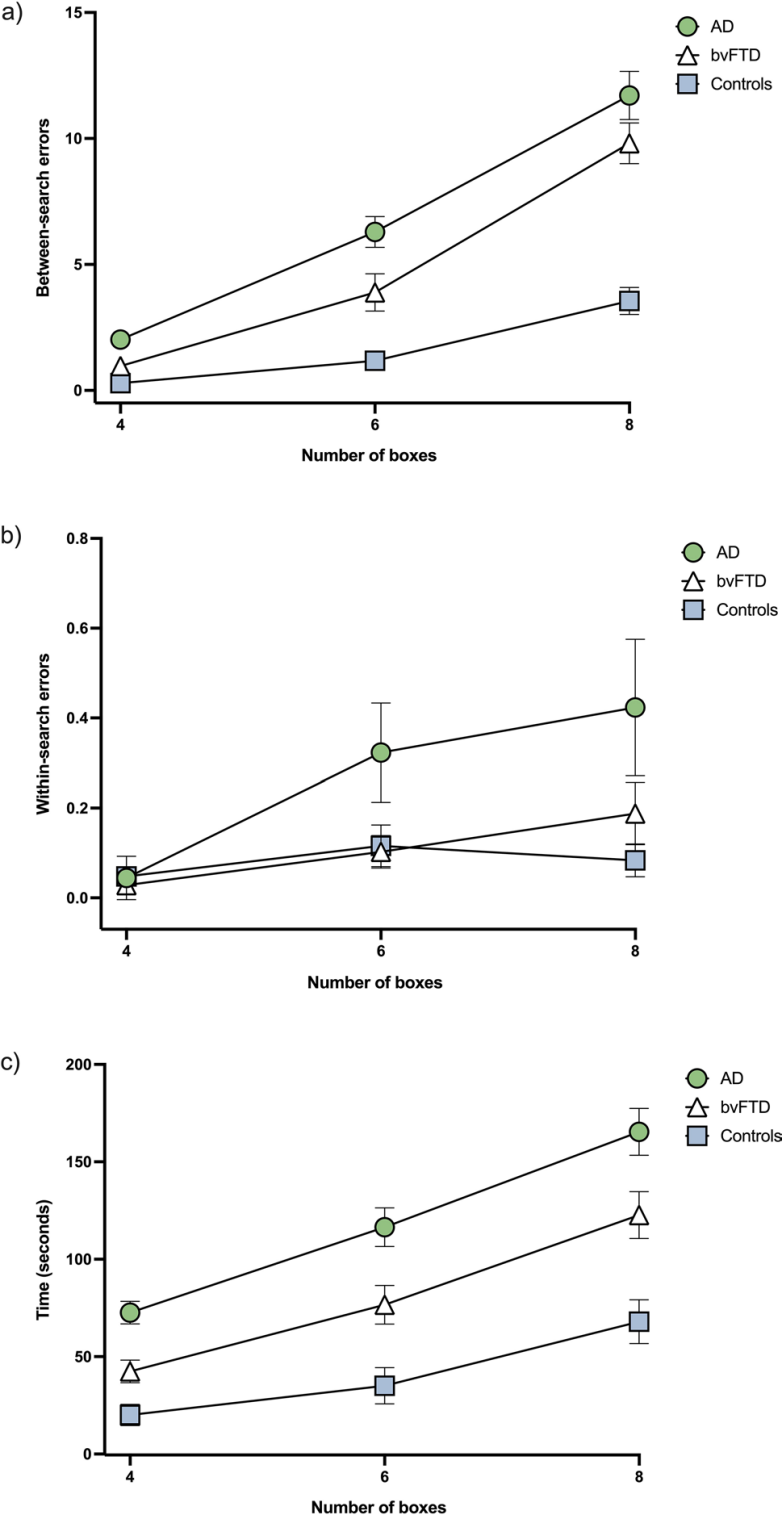
**a** Between-search errors, **b** within-search errors, and **c** time scores (in seconds) on the Box Task (means and standard error of the mean) in behavioural variant frontotemporal dementia (bvFTD), Alzheimer’s disease (AD) patients, and healthy controls. Higher scores represent worse performance. *Between-* and *within-search error* values are derived from the generalised estimating equations modelled data. These values are the predicted means over two trials at each set size (i.e., box size). The *time* values are raw mean scores derived from the repeated measures ANOVA. The *strategy* (i.e., normalised Levenshtein edit distance) score is displayed in Supplementary Fig. 1. The *between-* and *within-search* error raw scores are presented as scatterplots in Supplementary Fig. 2

A significant main effect of group was found for between-search errors (*p* < 0.001) (Table 2; Fig. 2a; Supplementary Table 2), driven by significantly more between-search errors in patients compared to controls overall (both *p* values < 0.001), as well as across all set sizes (4-box, *p* values ≤ 0.003; 6-box, *p* values < 0.001; 8-box, *p* values < 0.001). Additional comparisons between the patient groups revealed disproportionate impairments in the AD group relative to the bvFTD group on the 4-box (*p* = 0.002) and 6-box (*p* = 0.012) set sizes but not on the 8-box set size (*p* = 0.130) (Table 2).

**Table 2:** Estimated means and post-hoc group comparisons of the between-search errors, within-search errors, time taken, and strategy score on the Box Task at each set size (i.e., 4, 6, and 8 boxes)

Number of boxes	AD	bvFTD	Controls	Post hoc test^a^
Between-search errors				
4	2.02 (0.27)	0.97 (0.20)	0.29 (0.1)	AD > bvFTD > Controls
6	6.29 (0.61)	3.89 (0.74)	1.18 (0.32)	AD > bvFTD > Controls
8	11.71 (0.96)	9.81 (0.80)	3.55 (0.54)	AD, bvFTD > Controls
Within-search errors				
4	0.04 (0.05)	0.03 (0.02)	0.05 (0.02)	–
6	0.32 (0.11)	0.10 (0.04)	0.12 (0.05)	–
8	0.42 (0.15)	0.19 (0.07)	0.08 (0.04)	–
Time (seconds)				
4	72.62 (5.79)	42.39 (5.79)	20.08 (5.41)	AD > bvFTD > Controls
6	116.46 (9.95)	76.68 (9.95)	35.04 (9.31)	AD > bvFTD > Controls
8	165.36 (12.03)	122.66 (12.03)	67.97 (11.25)	AD > bvFTD > Controls
Strategy (Levenshtein edit distance)				
4	0.68 (0.04)	0.70 (0.03)	0.59 (0.03)	–
6	0.84 (0.02)	0.80 (0.02)	0.71 (0.02)	AD, bvFTD > Controls
8	0.80 (0.02)	0.81 (0.01)	0.70 (0.02)	AD, bvFTD > Controls

Values are the means (of two trials) ± standard error of the mean. The between-search error, within-search error, and strategy values (and post hoc group comparisons) are derived from the generalised estimating equations modelled data. The time values (and post hoc group comparisons) are derived from the repeated measures mixed model analysis of variance (ANOVA)

^a^Benjamini-Hochberg corrected

Within-group comparisons revealed all groups made more between-search errors as set size increased (all *p* values ≤ 0.001) (Supplementary Table 6).

### Within-search errors

No significant group differences were found for within-search error scores across all set sizes (all *p* values ≥ 0.030; not significant after Benjamini–Hochberg corrections) (Table 2; Fig. 2b; Supplementary Table 3). Within-group investigations revealed that the AD group made fewer errors at the 4-box set size than at the 6-box and 8-box set sizes (*p* values ≤ 0.007) (Supplementary Table 6). No significant differences were observed for the other groups (all *p* values ≥ 0.044; not significant after Benjamini–Hochberg corrections).

### Time to complete the task

Both the bvFTD and AD groups were slower to complete the task relative to the control group across all set sizes (all *p *values ≤ 0.006) (Table 2 and Fig. 2; see also Supplementary Table 5). In addition, AD patients took significantly longer to complete the task than the bvFTD patients (across set sizes and overall; *p* values ≤ 0.014). All groups took longer to complete the task as the set size increased (time duration for all groups: 4 boxes < 6 boxes < 8 boxes, all *p* values < 0.001) (Supplementary Table 6).

### Search path strategy (normalised Levenshtein edit distance score)

The normalised Levenshtein edit distance score provides an index of search path strategy efficiency. Relative to controls, search path strategy scores were higher (i.e., less efficient) for patients during overall task completion (both *p* values < 0.001) and across all set sizes (4-box, *p* values ≤ 0.003; 6-box, *p* values < 0.001; 8-box, *p* values < 0.001) (Table 2; Supplementary Fig. 1; Supplementary Table 4). No significant differences were present between patient groups (*p* values ≥ 0.621).

Task difficulty was also found to influence search path strategy. Irrespective of group, search path strategy was better at lower levels of task difficulty (i.e., 4-box vs 6-box set size; all *p* values < 0.003; 4-box vs 8-box set size; all *p* values ≤ 0.002), with no difference between the higher levels (i.e., 6-box vs 8-box *p* ≥ 0.111) (Supplementary Table 6).

### Contribution of visuospatial and non-verbal abilities to the Box Task 6-box set size performance

**Fig. 3:**
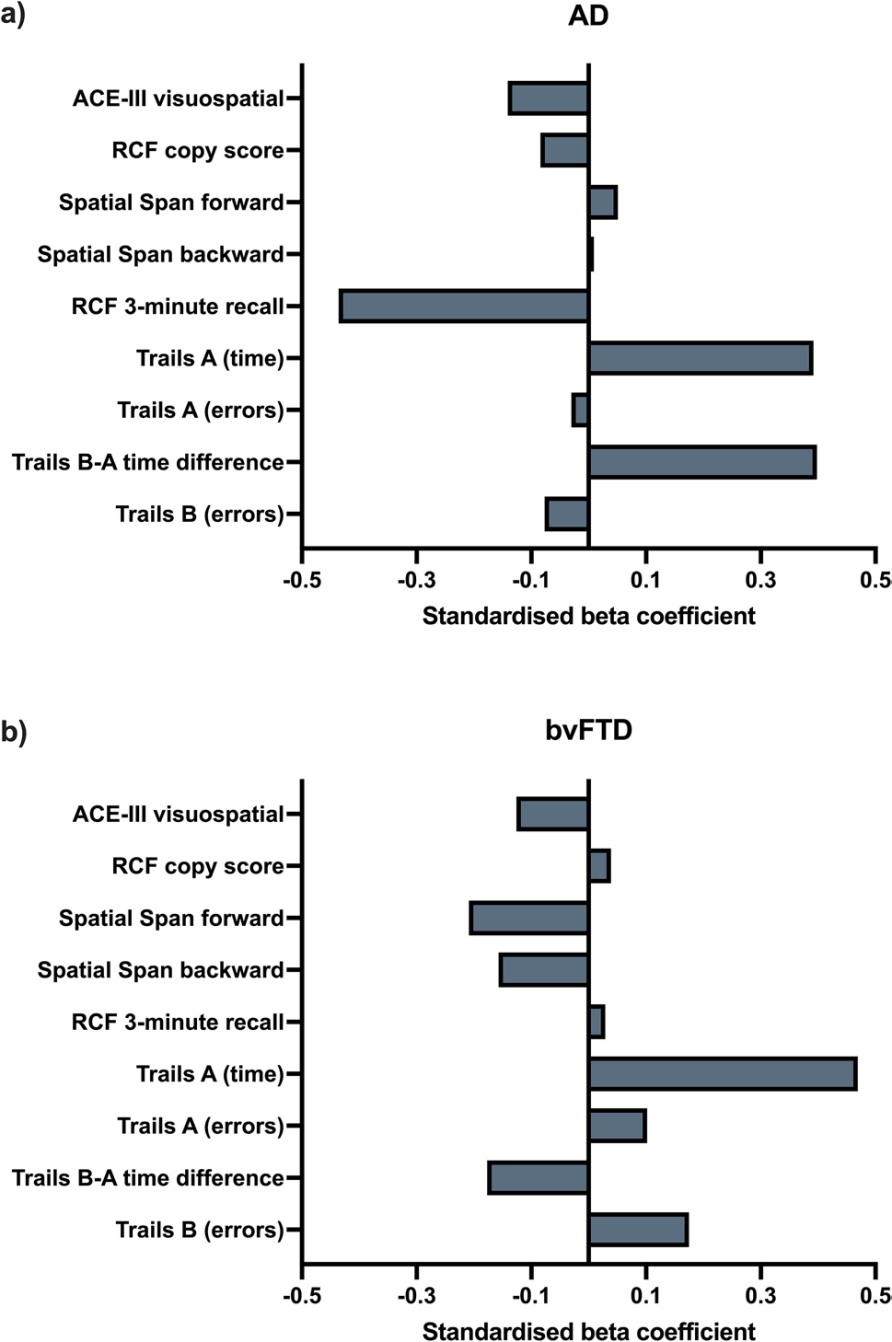
Standardised beta coefficient values of the **a** AD and **b** bvFTD mixed regression models. Standardised coefficient values indicate the amount by which the dependent variable changes if/when the Box Task 6-box set size between-search error (i.e., independent variable) changes by one unit, keeping all other independent variables constant. Estimated values are displayed in Supplementary Tables 7 and 8

Separate mixed regression models demonstrated that conventional visuospatial and non-verbal abilities (as measured by the ACE-III visuospatial subdomain, Spatial Span, RCF, and Trails A and B tests) were significant predictors of between-search error performance at the 6-box set size in AD (full model *R*^2^ = 0.511) and bvFTD (full model *R*^2^ = 0.593). Standardised beta coefficient values for each mixed regression model revealed distinct profiles across groups (Fig. 3; Supplementary Tables 7 and 8). In the AD group, visuospatial episodic memory (RCF 3-min recall) significantly predicted between-search error performance (*p* = 0.035) with the suggestion of a non-statistically significant trend for attention (Trails A time) (*p* = 0.070).

By contrast, in the bvFTD group, visuospatial episodic memory (RCF 3-min recall) did not significantly predict error performance (*p* = 0.871). Notably, however, attention (Trails A time) was on the threshold of significance (*p* = 0.053). Interestingly, lower Trails B-A time (i.e., an index of executive functioning) standardised beta coefficient value was related to *increased* between-search errors on the Box Task.

### Discriminating between bvFTD and AD based on Box Task and neuropsychological performance

**Fig. 4:**
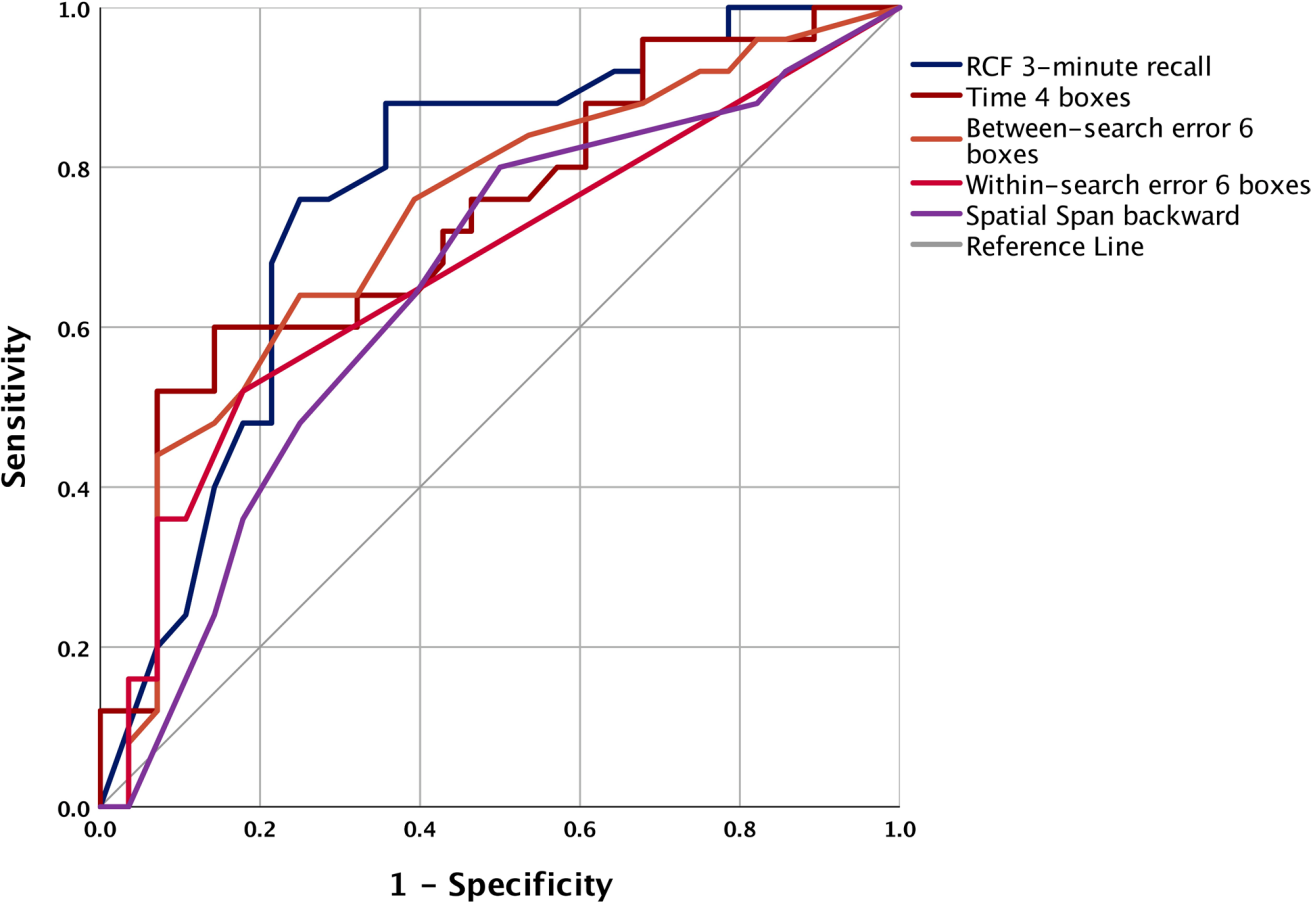
Areas Under the Receiver Operator Characteristic Curves (AUC) demonstrating that the RCF 3-min recall score (AUC = .771), Box Task 4-box time score (trial 2; AUC = .759) and Box Task 6-box between-search error score (average of trials 1 and 2; AUC = .747) are the three most sensitive and specific tests at classifying AD from bvFTD patients, relative to all other measures (Supplementary Table 9). Notably, most Box Task variables demonstrated larger AUCs than many conventional neuropsychological measures. The Box Task error and time scores were reversed for this figure (i.e., a higher score [more errors, longer time] is more indicative of AD than bvFTD)

The AUCs demonstrated that visual episodic memory performance (RCF 3-min recall; AUC 0.771), Box Task 4-box set size time (AUC 0.759), and Box Task 6-box set size between-search error performance (AUC 0.747) were the three most effective measures at discriminating bvFTD from AD, whereby AD performance was consistently worse than bvFTD on these measures (Fig. 4, Supplementary Table 9). Notably, the Box Task error (between-search: 4- and 6-box set sizes; within-search: 6-box set size) and time (all set sizes) measures were broadly more accurate than other conventional neuropsychological measures at differentiating bvFTD from AD (all AUCs > 0.700: 5 Box Task measures, 3 conventional neuropsychology measures). Finally, the AUCs largely confirmed that the AD group experienced greater learning and memory difficulties than the bvFTD group (e.g., Box Task error measures, Spatial Span, RCF, ACE-III memory subdomain).

### Classifying AD and bvFTD patients using the Box Task error measures

**Fig. 5:**
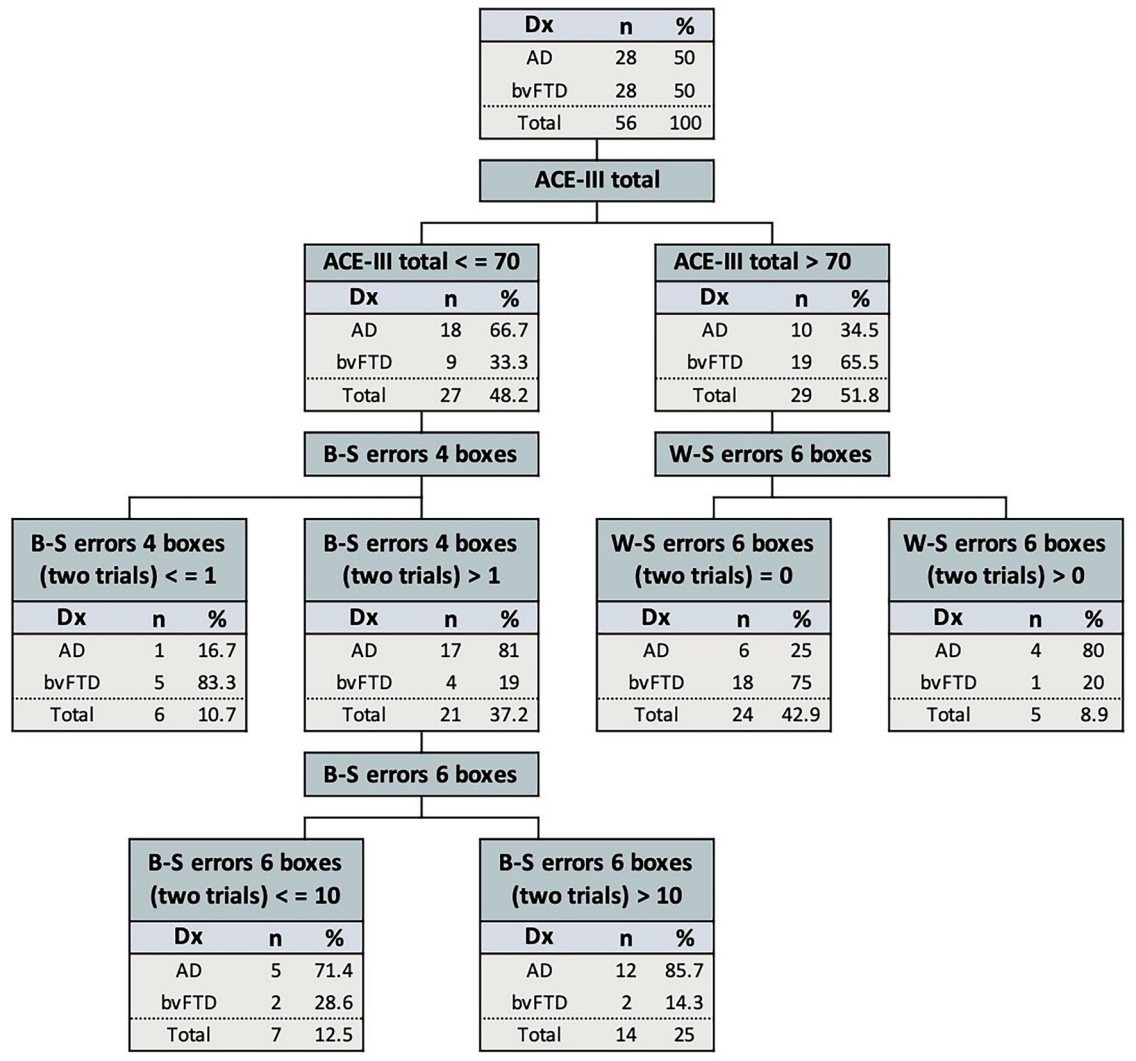
Chi-square automatic interaction detector (CHAID) decision tree to classify bvFTD versus AD patients based on their ACE-III Total and Box Task between- and/or within-error performance scores. The optimal cut-off values are the sum of trial 1 and 2. ACE-III: Addenbrooke’s Cognitive Examination-III; B-S errors: between-search errors; W-S errors: within-search errors; 4 boxes: 4-box set-size; 6 boxes: 6-box set-size

The CHAID demonstrated that Box Task between- and within-search error scores accurately classified 75% of AD patients (21/28 correctly classified) and 82.1% of bvFTD patients (23/28 correctly classified) (overall percent correct: 78.6%) after taking into account the ACE-III Total score as an index of overall cognitive impairment (≤ 70 vs > 70; n.b., first variable forced in the model). The CHAID model risk estimate was 0.214, indicating a 21% “risk” of misclassifying a patient (CHAID model standard error: 0.055; Fig. 5). For ACE-III scores above 70, the presence of one or more within-search errors (sum of Trials 1 and 2) at the 6-box set size was indicative of AD. For ACE-III scores 70 and below, between-search errors on the 4-box condition became the relevant error score, whereby the presence of 2 or more such errors was strongly suggestive of AD.

## Discussion

Mounting evidence indicates that episodic memory is impaired in bvFTD [3, 5, 7]. Using a novel, computerised test, the Box Task, we demonstrate that these impairments extend to the domain of visuospatial working memory. Overall, bvFTD patients displayed significant visuospatial working memory impairment across multiple levels of task difficulty. These impairments, however, were less severe than that observed in AD. Our findings suggest that visuospatial working memory deficits may need to be recognised as a relevant clinical feature of bvFTD. Moreover, our findings indicate that the Box Task offers potential diagnostic value in differentiating bvFTD from AD.

Our main finding was the presence of marked visuospatial working memory disturbances in bvFTD. Relative to controls, the bvFTD group displayed significantly more between-search errors on the Box Task across all levels of task difficulty. Between-search errors involve revisiting a location where an object has already been found and are suggested to reflect a breakdown in binding item and location information, as well as in basic processes such as object recognition and spatial-location processing [17]. Importantly, we found evidence of between-search impairment even at the lowest level of task difficulty (4-box set size), indicating a core disturbance of basic visuospatial memory processes (e.g., lower-load encoding and retention) that are not attributable to task demands. As task difficulty increased (i.e., at the 6- and 8-box set sizes), bvFTD patients made disproportionately more between-search errors than controls—likely reflecting a cumulative breakdown across multiple cognitive processes. In contrast, within-search performance, which requires comparatively lower cognitive demands [17], did not differ significantly among groups. This suggests that the between-search error measure of the Box Task elicits an optimal level of cognitive challenge and complexity compared to the within-search error measure. Taken together, our findings support the view that visuospatial working memory disruption is pervasive in bvFTD [10, 14] and suggest that the current clinical conception of bvFTD [4] may need revision to account for multidimensional memory disturbances spanning short-term, working, and episodic domains [1, 3, 6, 31, 32].

With respect to the AD group, visuospatial working memory was, unsurprisingly, severely compromised. Consistent with prior studies [18, 19], AD patients displayed significantly more between-search errors on the Box Task across all set sizes compared to controls. When compared with the bvFTD group, the AD group displayed significantly more between-search errors on the lower load 4- and 6-box set sizes but were not found to differ on the more cognitively demanding 8-box set size. These findings indicate that performance disparities between bvFTD and AD patients on the Box Task are most pronounced on visuospatial working memory tasks of low-to-moderate difficulty, and in patients with mild-to-moderate stages of dementia (disease duration of 3–5 years). Once task demands exceed a critical threshold (in this case the 6-box set size), patient groups display comparable error levels, suggesting the Box Task may be most useful in dementia diagnostics at lower to medium levels of cognitive demand [19]. Collectively, our Box Task and neuropsychological test findings reinforce the notion that alterations in visuospatial processing and memory dysfunction are core characteristics of AD [7, 11–13].

Visuospatial working memory encompasses a complex interplay of various processes, including perception, attention, executive functions, encoding, and short-term storage [33–36]. In bvFTD, impaired working memory is theorised to arise from frontal executive dysfunction, involving aspects such as sustained attention, mental flexibility, and response inhibition [10, 14]. Surprisingly, our mixed regression analysis of the Box Task 6-box set size between-search error profiles in bvFTD revealed that disruptions in higher-level executive functions (e.g., Trails Making Test B time) did not significantly predict Box Task error performance. Similarly, characteristics of short-term, working, and episodic memory (Spatial Span, RCF recall) did not significantly predict Box Task errors in bvFTD. Instead, attention (Trails A time) was on the threshold of significance in predicting task performance. In contrast, in the AD group, we had initially predicted that compromised Box Task performance would stem from a short-term capacity problem, encompassing difficulties in encoding, binding, and retaining information [10, 14, 19, 37, 38]. In keeping with this view, our regression analyses revealed that episodic memory impairment (i.e., RCF 3-min recall) significantly predicted Box Task performance in AD. Additionally, attention (Trails A time) showed a notable trend in its association with task performance. In sum, the observed deficits in visuospatial working memory in both bvFTD and AD appear to reflect the breakdown of distinct, rather than common, cognitive mechanisms [10, 14]. Further investigation, including imaging studies exploring underlying neural substrates, is needed to better understand this topic [6, 9, 39].

To evaluate the diagnostic utility of the Box Task within the context of neuropsychological testing, ROC analyses identified three key measures that accurately distinguished between bvFTD and AD patients: Box Task (6-box between-search error, 6-box within-search error, and 4-box time), RCF 3-min recall, and the ACE-III memory subdomain. Conceptually, the high sensitivity of two visuospatial memory tests and one verbal memory test is not surprising, given the disproportionate impact of AD pathology on visuospatial ability and memory [7, 12, 13, 19]. Tests that evaluate these cognitive skills simultaneously would compound the challenge for AD patients [7, 12, 13, 19]. From a clinical standpoint, accounting for disease severity and overall cognitive capacity becomes imperative when considering a bvFTD versus AD differential diagnosis [32, 40, 41]. To this end, we investigated the effectiveness of the Box Task error measures while considering overall cognitive ability. By combining between-search and within-search errors on the Box Task with the ACE-III total score, our decision tree accurately classified 82% of bvFTD patients and 75% of AD patients. These findings underscore the Box Task’s potential in accurately distinguishing between bvFTD and AD, particularly when considering disease severity and its multiple embedded metrics. Further research, including the establishment of normative data on the Box Task, is required before this test becomes suitable for clinical application.

Finally, focusing on the qualitative aspects of Box Task performance in our cohort, the time taken to complete the task emerged as a strong predictor of AD. Patients with AD consistently spent more time completing the task compared to bvFTD patients across all box set sizes, aligning with clinical observations where AD patients typically exhibit hesitancy and slower performance, while bvFTD patients tend to be more impulsive [10]. We did not, however, observe any discernible differences between the two groups in their strategy approach to the task, as measured by the Levenshtein distance score. It is important to note that the search strategy is likely multidimensional and warrants further investigation. Taken together, our findings contribute to a more nuanced qualitative understanding of these conditions.

While preliminary, our findings carry implications for their application in the clinical setting. Accurate differentiation of clinically probable bvFTD from AD remains challenging, particularly in patients where MRI or corroborative biomarkers are not available [2, 13, 32, 40, 42]. Our study indicates that including targeted tests of visuospatial working memory (Box Task) alongside episodic memory measures (ACE-III memory subscale; RCF 3-min Recall) as part of the diagnostic workup may facilitate the early and accurate identification of these syndromes. Notably, the Box Task offers several advantages over traditional neuropsychological tests: it is brief, easy to administer without extensive training, and measures various cognitive processes (e.g., visuospatial functioning, working memory) by assessing different error types and levels of impairment. Moreover, qualitative aspects such as task completion time and search path strategy provide insights into potential underlying mechanisms contributing to task failure. Nonetheless, it will be important to replicate these findings in a larger cohort and to establish appropriate cut-off scores to ensure the Box Task test is suitable for clinical use.

Similarly, we note several methodological limitations that warrant consideration. First, to increase study power, we combined sporadic and familial bvFTD patients into a single group; however, we acknowledge that memory profiles in this syndrome may vary based on the underlying genetic profile [43]. Second, our study did not include cases with possible bvFTD or atypical variants of FTD or AD. As such, the clinical utility of the Box Task in distinguishing these clinical phenotypes is unknown. The decision to focus on probable bvFTD and typical (i.e., amnestic) AD was grounded in the perceived clarity of their neuropsychological profiles according to current diagnostic criteria. As such, our findings were able to directly challenge current assumptions of the canonical neuropsychological profile of bvFTD. Third, we were not able to include independent biomarker information or confirmation of underlying disease pathology in our study participants. While we recognise the role of biological biomarkers in shaping the future of dementia diagnosis [42], we note that the current diagnostic criteria for bvFTD continue to guide clinical practice, and neuropsychological evaluations for dementia diagnosis are often conducted without access to biomarker data. Fourth, while our AD group was age-matched to the bvFTD group, our AD cohort may primarily represent younger-onset AD (mean age of AD cohort: 66; age range: 53–78), potentially limiting the generalisability of our findings across the entire age spectrum of AD. Fifth, while we used well-accepted measures of memory function, it is not clear how these disturbances relate to subjective experiences of (working) memory lapses in daily life [44]. Further investigation is necessary to arrive at a comprehensive understanding of the relationship between memory impairments as revealed by objective test measures and how these impact the patient in their daily life. Finally, given that apathy is pervasive in bvFTD [45, 46], it will be important to clarify how non-cognitive changes, such as loss of goal-directed behaviour, potentially influence task performance.

In conclusion, this study offers important insights into the visuospatial memory profiles of bvFTD and AD. Our findings reveal significant visuospatial working memory impairment in bvFTD, albeit of lesser severity compared to disease-matched AD patients. Assessments targeting visuospatial memory, including the Box Task, demonstrate promising discriminatory capabilities between bvFTD and AD. Overall, this research underscores the clinical significance of visuospatial memory evaluation in effectively distinguishing between bvFTD and AD.

### Supplementary Information

Below is the link to the electronic supplementary material.

Supplementary file1 (PDF 476 KB)

## Data Availability

The datasets generated during and/or analysed during the current study are available from the corresponding author upon reasonable request. No part of the study procedures or analyses were preregistered prior to the research being undertaken. The Box Task is freely available at https://roykessels.nl/tests-and-software/box-task. The Addenbrooke’s Cognitive Examination-Third edition (ACE-III) is freely available at frontierftd.org. Legal copyright restrictions prevent public archiving of the other neuropsychological tests used in this research. These materials can be obtained from the copyright holders in the cited references. The SPSS syntax used for this project has been made available for review on the Open Science Framework website (https://osf.io/wsqpy/).
