# Correction to: Visuospatial working memory in behavioural variant frontotemporal dementia: a comparative analysis with Alzheimer's disease using the box task

**DOI:** 10.1007/s00415-024-12483-1

**Published:** 2024-06-21

**Authors:** David Foxe, Muireann Irish, James Carrick, Sau Chi Cheung, Her Teng, James R. Burrell, Roy P. C. Kessels, Olivier Piguet

**Affiliations:** 1 School of Psychology, The University of Sydney, Sydney, Australia; 2 Brain and Mind Centre, The University of Sydney, 94 Mallett St, Sydney, NSW 2006 Australia; 3 Neuropsychology Unit, Royal Prince Alfred Hospital, Sydney, Australia; 4 Concord Medical School, The University of Sydney, Sydney, Australia; 5 Donders Institute for Brain, Cognition and Behaviour, Radboud University, Nijmegen, The Netherlands; 6 Vincent Van Gogh Institute for Psychiatry, Venray, The Netherlands; 7 Radboud University Medical Center, Radboudumc Alzheimer Center, Nijmegen, The Netherlands

**Correction to: Journal of Neurology** 10.1007/s00415-024-12406-0

In the original version of this article, there was an error in the reference citation. 25 was replaced as 24. This means that all citations from 25 onwards are systematically one short what they should be (i.e. citation 25 should be 26, 26 should be 27, 27 should be 28 etc.) and the first instance of citation 22 was not hyperlinked.

The original article has been corrected.

