# Supplementary material for: Visuospatial working memory in behavioural variant frontotemporal dementia: a comparative analysis with Alzheimer's disease using the box task
Source: J Neurol. 2024 May 7;271(8):4852–63. doi: 10.1007/s00415-024-12406-0 (PMC11319587; doi:10.1007/s00415-024-12406-0)
Supplement: Supplementary file 1 — Supplementary file1 (PDF 476 KB) [file 415_2024_12406_MOESM1_ESM.pdf]

Supplementary Material

Visuospatial Working Memory in Behavioural Variant Frontotemporal Dementia: A Comparative Analysis with Alzheimer's Disease using the Box Task

David Foxe*, Muireann Irish, James Carrick, Sau Chi Cheung, Her Teng, James R. Burrell, Roy P.C. Kessels, Olivier Piguet

***Corresponding author:** David Foxe, Brain & Mind Centre, The University of Sydney, 94 Mallett St, Sydney NSW 2006, Australia

Email: david.foxe@sydney.edu.au

Methods

The chi-square automatic interaction detector (CHAID) method was used to determine the optimal cut-off Box Task performance scores to discriminate bvFTD from AD. To derive a relatively simple decision tree (and as the main measures of the Box Task are the errors scores), the between- and within-search errors (only) at all set sizes were included in the model. Important to the rationale of this analysis is the awareness that the AD and bvFTD patients in this study demonstrate variable overall cognitive function. Accordingly, from a clinical perspective, we deemed it was important to consider the patient's overall cognitive ability before forming clinically relevant cut-off points on the Box Task. We therefore decided to include the ACE-III total score (a measure of overall cognitive ability) as the first forced variables in the model. For the remaining Box Task error variables, the CHAID automatically selected each variable based on its own merit.

Results

Performance on neuropsychological tests

Significant group differences were observed on all neuropsychological tests, with the exception of the error measure on Trail Making Test A ($H(2) = 1.176$, $p = .555$; Supplementary Table 1). Post hoc tests revealed characteristic cognitive profiles of each dementia subtype. Relative to controls, the AD and bvFTD groups displayed impairments on all tests of attention and executive functioning (ACE-III attention and fluency, Digit Span forward and backward, Trails A and B time; Trails B errors: AD, all *p*-values <.001; bvFTD, all *p*-values $\leq$ .034), verbal memory (ACE-III memory: AD and bvFTD, both *p*-values < .001) and language (ACE-III language, SYDBAT tests: AD, all *p*-values $\leq$ .03; bvFTD, all *p*-values < .007).

Direct comparison of the patient groups revealed the AD group was more impaired than the bvFTD group on verbal memory (ACE-III memory, $p = .002$) and were slower on measures of attention (Trail A time, $p = .038$) and executive functioning (i.e., rapid set-shifting) (Trails B time, $p = .040$). There were no significant group differences on other measures of attention and executive function and

language (ACE-III attention, fluency, and language, Digit Span forward and backward, SYDBAT subtests: all *p*-values $\geq$ .088).

Supplementary Table 1. Neuropsychological profiles of study participants

	AD	bvFTD	Controls	Group effect (F or H)	*p*	Post hoc test (Sidak Corrected)
**Attention, short-term and working memory, executive functioning**						
ACE-III attention (18)	12.5 (3.3)	14.1 (3.5)	17.5 (0.8)	26.203	< .001	AD, bvFTD < Controls
Digit Span forward (16)	8.8 (1.5)	9 (2.3)	11.3 (2.3)	12.907	< .001	AD, bvFTD < Controls
Digit Span backward (14)	4.4 (1.6)	4.6 (1.9)	7.7 (2.1)	29.23	< .001	AD, bvFTD < Controls
Trails A (secs)	73.3 (43.5)	54.3 (22.1)	30.9 (7.8)	17.345	< .001	AD > bvFTD > Controls
Trails A errors	0.2 (0.4)	0.4 (1)	0.1 (0.3)	1.176^	.555	—
Trails B (secs)	229.5 (118)	164.5 (92.7)	75.9 (28.5)	22.286	< .001	AD > bvFTD > Controls
Trails B errors	1.7 (1.5)	1.6 (2)	0.4 (0.8)	14.209^	< .001	AD, bvFTD > Controls
ACE-III fluency (14)	7.9 (2.8)	6.8 (3.1)	12.1 (1.5)	37.169	< .001	AD, bvFTD < Controls
**Verbal memory**						
ACE-III memory (26)	12.7 (3.9)	16.4 (5.4)	24.7 (1.4)	76.763	< .001	AD < bvFTD < Controls
**Language**						
ACE-III language (26)	22.7 (2.6)	22.4 (3)	25.4 (1)	15.589	< .001	AD, bvFTD < Controls
SYDBAT naming (30)	22 (4.4)	21.6 (4.5)	27 (2.1)	18.94	< .001	AD, bvFTD < Controls
SYDBAT repetition (30)	29.2 (1.7)	28.6 (1.9)	29.9 (0.3)	11.340^	.003	AD, bvFTD < Controls
SYDBAT comprehension (30)	26.3 (2.4)	26.2 (3.5)	29.3 (1.1)	16.928	< .001	AD, bvFTD < Controls
SYDBAT semantic (30)	24.6 (3.3)	24.3 (3.5)	28.4 (1)	33.898^	< .001	AD, bvFTD < Controls

Notes: Values are mean ± standard deviation. ^ = Kruskal-Wallis test with Dunn-Bonferroni correction. Abbreviations: ACE-III: Addenbrooke's Cognitive Examination–Third edition; AD: Alzheimer's disease; bvFTD: behavioural variant frontotemporal dementia; SYDBAT: The Sydney Language Battery; Trails: Trail Making Test. Number of missing values: Trails A (secs) = 1 AD; Trails A errors = 1 AD, 1 bvFTD; Trails B (secs) = 11 AD, 6 bvFTD; Trails B errors = 9 AD, 5 bvFTD; SYDBAT naming = 10 bvFTD, 1 control; SYDBAT repetition = 1 AD, 10 bvFTD; SYDBAT comprehension = 1 AD, 10 bvFTD; SYDBAT semantic = 1 AD, 10 bvFTD.

Supplementary Table 2. Generalised estimating equations model of the Box Task between-search errors

	Wald Chi-Square	df	*p*-value
Intercept	114.687	1	< .001
Diagnosis	48.902	2	< .001
Box level (nested under diagnosis)	516.463	6	< .001
Box trial (nested under diagnosis)	18.369	3	< .001

Notes, Box level = the number of boxes that the participant has to search (i.e., 4, 6 or 8 boxes). Box trial = the two trials within each box level (i.e., set size).

Supplementary Table 3. Generalised estimating equations model of the Box Task within-search errors

	Wald Chi-Square	df	*p*-value
Intercept	19.725	1	< .001
Diagnosis	3.552	2	.169
Box level (nested under diagnosis)	19.827	6	.003
Box trial (nested under diagnosis)	1.296	3	.730

Notes, Box level = the number of boxes that the participant must search (i.e., 4, 6 or 8 boxes). Box trial = the two trials within each box level (i.e., set size).

Supplementary Table 4. Generalised estimating equations model of the Box Task strategy (i.e., normalised Levenshtein edit distance) scores

	Wald Chi-Square	df	*p*-value
Intercept	5448.027	1	< .001
Diagnosis	21.114	2	< .001
Box level (nested under diagnosis)	46.867	6	< .001
Box trial (nested under diagnosis)	6.84	3	.077

Notes, Box level = the number of boxes that the participant must search (i.e., 4, 6 or 8 boxes). Box trial = the two trials within each box level (i.e., set size).

Supplementary Table 5a and 5b. Repeated measures mixed model analysis of variance (ANOVA) of the Box Task time scores

5a. Tests of between-subject effects

	Type III Sum of Squares	df	Mean Square	F	Sig.	Partial Eta Squared
Intercept	1679487.319	1	1679487.319	266.398	> .001	.758
Diagnosis	267135.326	2	133567.663	21.186	> .001	.333
Error	535876.99	85	6304.435			

5b. Tests of Within-Subjects Contrasts

	Average_time	Type III Sum of Squares	df	Mean Square	F	Sig.	Partial Eta Squared
Average_time	Linear	237647.342	1	237647.342	237.436	> .001	.736
	Quadratic	1958.025	1	1958.025	4.306	.041	.048
Average_time * Diagnosis	Linear	16269.34	2	8134.67	8.127	.001	.161
	Quadratic	415.123	2	207.561	.456	.635	.011
Error (Average_time)	Linear	85075.583	85	1000.889			
	Quadratic	38652.339	85	454.733			

Notes, Average_time = the mean time of trial 1 and trial 2 at each set size (i.e., 6-box, 8-box) with the exception of the 4-box set size: this time value is the Trial 2 time (only) score.

Supplementary Table 6. Post hoc set size (i.e., box level) comparisons of between-search errors, within-search errors, strategy score, and time in each group

	Group	Post hoc test (Benjamini-Hochberg corrected)
Between-search errors		
	AD	4 boxes < 6 boxes < 8 boxes
	bvFTD	4 boxes < 6 boxes < 8 boxes
	Controls	4 boxes < 6 boxes < 8 boxes
Within-search errors		
	AD	4 boxes < 6 boxes, 8 boxes
	bvFTD	-
	Controls	-
Strategy score		
	AD	4 boxes < 6 boxes, 8 boxes
	bvFTD	4 boxes < 6 boxes, 8 boxes
	Controls	4 boxes < 6 boxes, 8 boxes
Time (seconds)		
	AD	4 boxes < 6 boxes < 8 boxes
	bvFTD	4 boxes < 6 boxes < 8 boxes
	Controls	4 boxes < 6 boxes < 8 boxes

Notes: AD = Alzheimer's disease; bvFTD = behavioural variant frontotemporal dementia; Strategy score = normalised Levenshtein edit distance score. The between-search error, within-search error, and strategy post hoc group comparisons are derived from the generalised estimating equations modelled data. The time post hoc group comparisons are derived from the repeated measures mixed model analysis of variance (ANOVA).

Supplementary Table 7. Variables contributing to Box Task 6 set-size between-search error performance in Alzheimer's disease (AD)

	B	S.E.	β	p	Pearson r	Partial
(Constant)	5.333	3.687		0.165		
ACE-III visuospatial	-0.135	0.237	-0.141	0.577	-0.285	-0.133
RCF copy score	-0.034	0.096	-0.084	0.726	-0.456	-0.084
Spatial Span forward	0.076	0.400	0.051	0.851	-0.251	0.045
Spatial Span backward	0.011	0.324	0.009	0.973	-0.358	0.008
RCF 3-minute recall	-0.443	0.194	-0.436	0.035*	-0.413	-0.475
Trails A (time)	0.030	0.015	0.392	0.070	0.413	0.413
Trails A (errors)	-0.250	1.851	-0.030	0.894	0.311	-0.032
Trails B-A time difference	0.015	0.011	0.398	0.214	0.349	0.291
Trails B (errors)	-0.198	0.740	-0.077	0.792	0.310	-0.063

Notes, β = estimated b-values for the model, Partial = partial correlation coefficient, Pearson r = Pearson correlation coefficient, S.E. = estimated standard error, β = beta standardised coefficients, * = $p < .05$.

Supplementary Table 8. Variables contributing to Box Task 6 set-size between-search error performance in behavioural variant frontotemporal dementia (bvFTD)

	B	S.E.	β	p	Pearson r	Partial
(Constant)	7.437	9.038		0.421		
ACE-III visuospatial	-0.351	0.582	-0.126	0.554	-0.537	-0.141
RCF copy score	0.023	0.167	0.039	0.892	-0.471	0.032
Spatial Span forward	-0.420	0.493	-0.209	0.405	-0.579	-0.197
Spatial Span backward	-0.271	0.563	-0.157	0.636	-0.580	-0.113
RCF 3-minute recall	0.017	0.106	0.029	0.871	-0.270	0.039
Trails A (time)	0.088	0.043	0.469	0.053	0.627	0.438
Trails A (errors)	0.413	0.656	0.102	0.537	0.086	0.147
Trails B-A time difference	-0.003	0.004	-0.177	0.467	0.213	-0.173
Trails B (errors)	0.395	0.425	0.175	0.365	0.422	0.214

Notes, β = estimated b-values for the model, Partial = partial correlation coefficient, Pearson r = Pearson correlation coefficient, S.E. = estimated standard error, β = beta standardised coefficients.

Supplementary Table 9. Areas Under the Receiver Operator Characteristic Curves (AUC) of all tests at classifying behavioural variant frontotemporal dementia (bvFTD) from Alzheimer's disease (AD) patients

Test	AUC	S.E.M.	Asymptotic Sig.
RCF 3-minute recall	0.771**	0.066	0.001
Box Task 4 boxes time	0.759**	0.065	0.001
Box Task 6 boxes between-search errors	0.747**	0.067	0.002
Box Task 6 boxes time	0.740**	0.067	0.002
Box Task 4 boxes between-search errors	0.728**	0.068	0.003
RCF copy time	0.723**	0.072	0.006
ACE-III memory	0.704**	0.073	0.009
Box Task 6 boxes within-search errors	0.702*	0.071	0.01
Spatial Span backward	0.682*	0.072	0.02
Trails B time	0.675	0.09	0.064
Box Task 8 boxes time	0.663	0.073	0.036
ACE-III visuospatial	0.658	0.074	0.043
Box Task 8 boxes within-search errors	0.643	0.074	0.066
ACE-III attention	0.636	0.076	0.08
Spatial Span forward	0.614	0.076	0.145
Trails A time	0.612	0.076	0.152
Box Task 8 boxes between-search errors	0.591	0.077	0.245
SYDBAT repetition	0.574	0.092	0.41
SYDBAT semantic	0.560	0.091	0.504
SYDBAT naming	0.544	0.090	0.625
RCF copy	0.533	0.081	0.682
Box Task 4 boxes strategy	0.530	0.078	0.700
SYDBAT comprehension	0.493	0.096	0.933
Within-search errors 4 boxes	0.491	0.078	0.909
ACE-III language	0.485	0.079	0.844
Box Task 8 boxes strategy	0.483	0.079	0.831
Box Task 6 boxes strategy	0.448	0.078	0.507
ACE-III verbal fluency	0.411	0.077	0.255

Notes: * $p < .05$; ** $p < .01$; S.E.M. = standard error of the mean. The Box Task variables are the average score of trial 1 and 2. The Box Task error and time scores Abbreviations: ACE-III: Addenbrooke's Cognitive Examination–Third edition; Letter fluency: Letters F, A, S. RCF: Rey Complex Figure; Spatial Span: Spatial Span from the Wechsler Memory Scale-Third Edition; SYDBAT: The Sydney Language Battery; Trails: Trail Making Test. Number of missing values: RCF copy score: 3 AD; RCF copy time: 3

AD, 1 bvFTD; RCF 3-minute recall: 3 AD; SYDBAT all subtests: 2 AD, 10 bvFTD; Trails A time: 1 AD; Trails B time: 11 AD, 6 bvFTD.

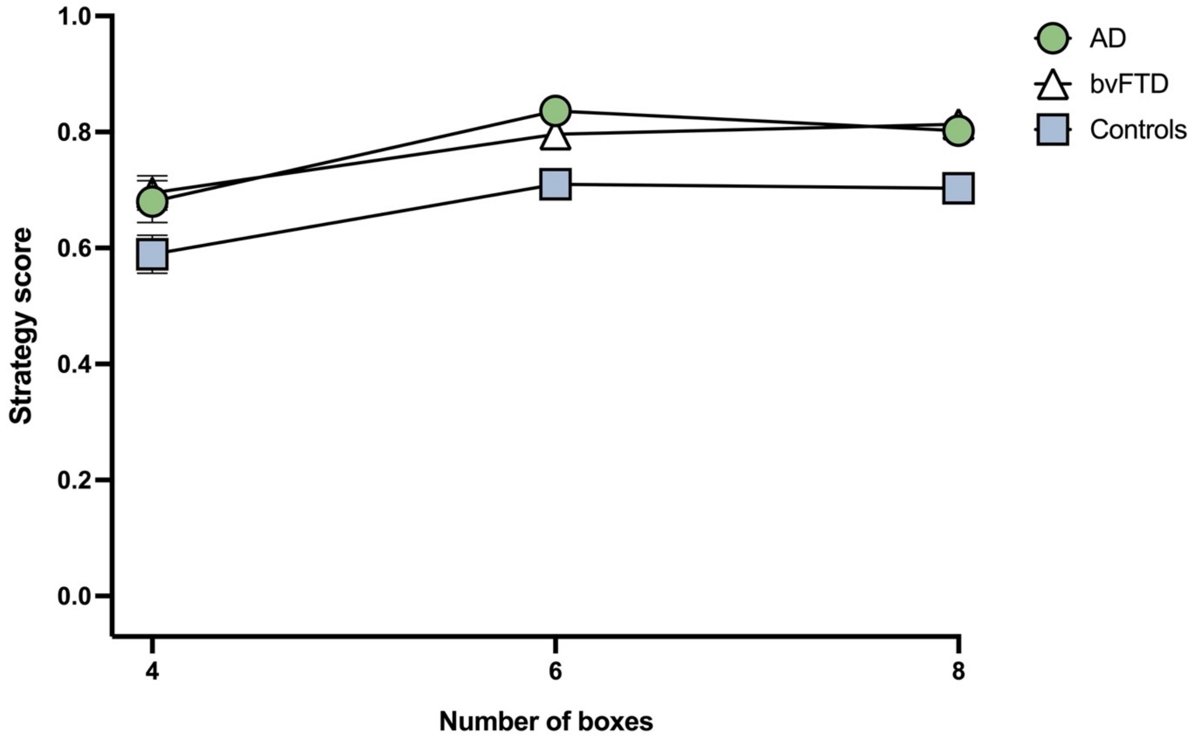

Supplementary Figure 1. Normalised Levenshtein edit distance (i.e., strategy) scores in behavioural variant frontotemporal dementia (bvFTD), Alzheimer's disease (AD) patients, and healthy controls. Notes: Values are derived from the generalised estimating equations modelled data. It is assumed lower distance scores denote a more proficient strategy to complete the task.

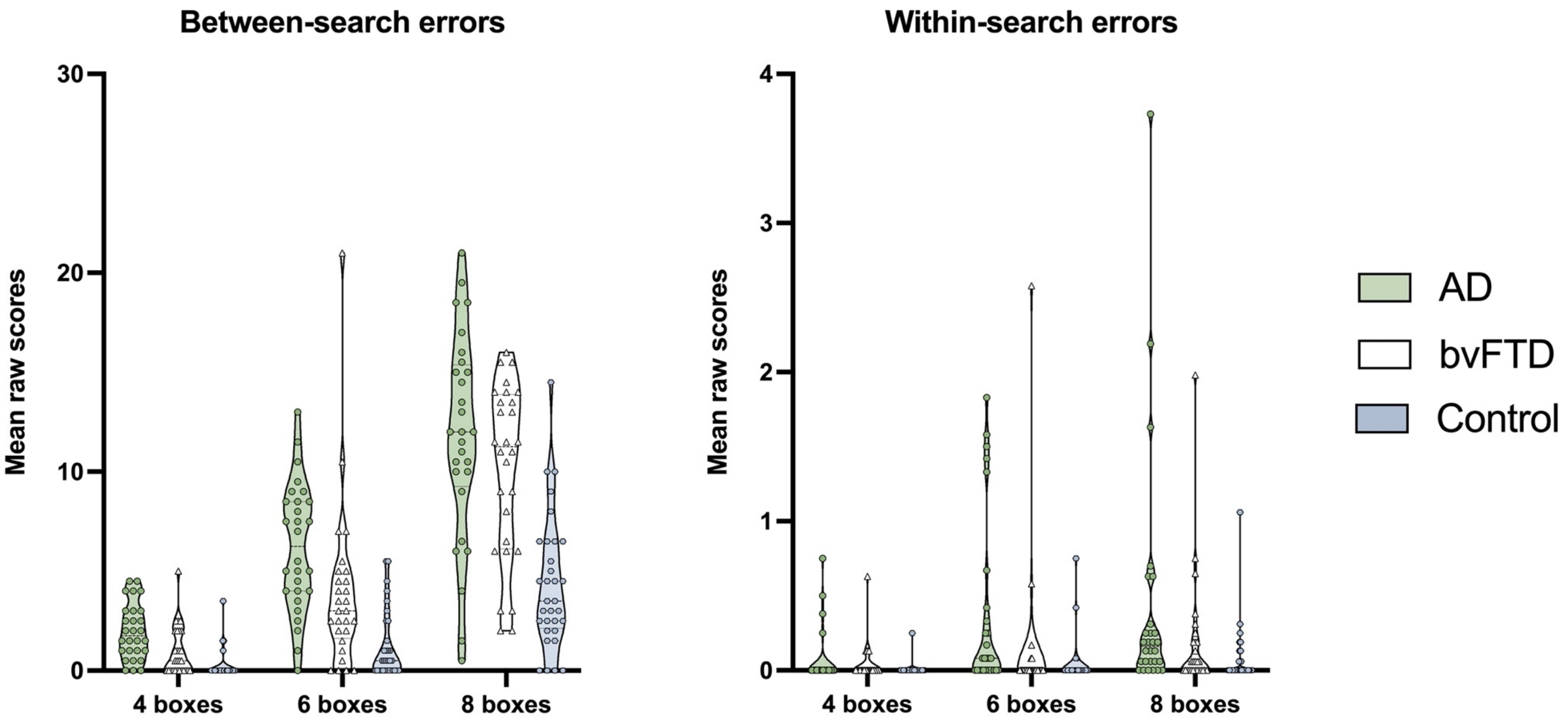

Supplementary Figure 2. Raw between-search and within-search error scores on the Box Task.

Note, scatterplots represent the participants mean error score over Trials 1 and 2.
